# Abomasal lesions found in postmortem examination of fattening Holstein-Friesian bulls from central Poland

**DOI:** 10.1007/s11259-024-10366-4

**Published:** 2024-03-28

**Authors:** Paulina Pyrek, Karol Witt, Monika Szpringiel, Bartłomiej Maria Jaśkowski

**Affiliations:** 1 Department of Internal Medicine and Clinic of Diseases of Horses, Dogs and Cats, Faculty of Veterinary Medicine, Wroclaw University of Environmental and Life Sciences, Grunwaldzki Sq. 47, Wrocław, 50-366 Poland; 2 Department of Production Animal Clinical Sciences (PRODMED), Faculty of Veterinary Medicine, Norwegian University of Life Sciences, Elizabeth Stephansens vei 15, Ås, 1430 Norway; 3 Department of Reproduction and Clinic for Farm Animals, Faculty of Veterinary Medicine, Wroclaw University of Environmental and Life Sciences, Grunwaldzki Sq. 49, Wrocław, 50-366 Poland

**Keywords:** Abomasum, Fattening bulls, Inflammation, Parasites, Poland, Ulcers

## Abstract

The health of the gastrointestinal tract in cattle is one of the factors influencing adequate nutrient absorption and body weight gain. This study aimed to evaluate the prevalence of lesions found in the abomasum of Polish fattening Holstein-Friesian bulls. A total of 149 abomasa from adult bulls from two voivodeships of central Poland were inspected for pathologicalanatomic position (displacement, volvulus), impaction, mucosal membrane lesions (inflammation, hyperplasia), and signs of parasite infestation(Moroccan leather, worm nodules) in one of the Polish abattoirs just after slaughter. Among all the examined males no lesions were observed in only 8 individuals. The most common findings were erosions (75.8%) and type 1 ulcerations (48.3%) of the body, pylorus, or both locations. Signs of parasite infestations were observed in 78.5% of the inspected animals. Significant differences correlated with age were found in the prevalence of healed ulcers, and between voivodeships in the prevalence of type 2 ulcers. The findings of this study indicate that most fattening bulls in Poland suffer from subclinical forms of abomasa inflammation (both diffuse and focal) and middle-large abomasum parasites invasions, which can influence the economics and profitability, as well as welfare, of livestock production.

**Supplementary Information:**

The online version contains supplementary material available at 10.1007/s11259-024-10366-4.

## Introduction

Diseases of the digestive system influence animal welfare and cattle productivity leading to economic losses and secondary health problems. Every year 10% of Polish cattle are slaughtered because of gastrointestinal and metabolic disorders (Pawlina et al. 2015). One of the reasons is abomasum diseases, but this problem is not widely known because of a lack of accurate data about their prevalence. In the field, *antemortem* diagnosis of the left or right displaced abomasum, abomasal volvulus, and impaction are possible but recorded mostly only in dairy cows (Kuiper 1991). Diagnosis of other abomasum disorders is limited as the first stages of the disease are difficult to observe during routine examination. Therefore, some of these diseases go unnoticed.

Among them, parasite invasion, inflammation, and hyperplastic lesions are the most likely to be missed. Abomasal ulcers and erosions are most often recorded during *postmortem* examination, as animals affected with small lesions are usually asymptomatic (Kuiper 1991). Abomasitis, hyperplastic lesions, and neoplasms do not play a key role in livestock production according to the low prevalence described in the literature (Fubini et al. 2018). Despite the prevalence of parasite invasions, regular monitoring of infestation intensity is not a widespread practice on farms. Most often, cattle are either treated seasonally, or no prophylaxes are used in herd management (Strydom et al. 2023).

In 2021, adult bulls accounted for 50.8% of all cattle slaughtered in Poland, which makes them an important part of Polish farms profitability (Statistics Poland 2022). Literature review shows that mortality due to digestive tract disorders is second only to respiratory problems in feedlot cattle (Nagaraja and Lechtenberg 2007). However, fattening males are usually not a group of concern in the case of abomasum disorders. This study aimed to evaluate if the problem of abomasum disorders exists in the population of fattening Holstein-Friesian (HF) bulls kept on Polish farms, regardless of the housing and feeding system applied.

## Materials and methods

### Animals and material

In total, the abomasa of 149 healthy Holstein-Friesian bulls aged 12–39 months were examined for two consecutive days in one of the largest abattoirs in central Poland. Individuals were sent for regular slaughter from two voivodeships: Greater Poland and Masovian (Table 1). Animals were divided into two age groups according to the division used in Polish national statistics: bulls younger than and older than 24 months (Table 2). Both groups were random, with no prior selection performed, and examined bulls accounted for 3% of the bulls and bullocks slaughtered at that time in both voivodeships. Abomasa were inspected directly after slaughter, during the evisceration process, each time by the same team of two veterinarians. The abomasa were cleaned in the evisceration sector, put in reverse to avoid cutting, and examined for macroscopic lesions in the mucosa, and signs of parasite infestation. More detailed information on individual bulls was obtained from the identification number given to each animal following slaughter, which correlated to bulls ear tag number (Table 1).

**Table 1:** Origin and age of examined Holstein-Friesian bulls (*n* = 149)

	Greater Poland	Masovian Voivodeship
Number of bulls examined	109	40
Number of farms involved	17	10
Age range in months (min-max)	12–39	18–22
Medium age in months ± SD	20.3 ± 4.61	24 ± 3.98

### Anatomic position and macroscopic lesions evaluation scale

The pathological anatomic position, discriminating between left (LDA) and right displacement (RDA) of the abomasum, as well as the presence of volvulus, were recorded during the evisceration process. The abomasum wall was checked for possible dilatation connected with the impaction. Lesions inspected on the mucous membrane were inflammation and hyperplasia, and their location in the different parts of the abomasum (fundus, body, pylorus) was noted (Budras and Wünsche 2003). Two types of inflammation were examined: focal (erosions, ulcers) and diffuse (abomasitis). Diffuse inflammation was detected when redness, hemorrhages, emphysema, and oedema of the mucous membrane on the large surface could be observed (Gelberg 2017). In this study, erosions, historically classified as type 1 ulcers, were distinguished and separated as superficial lesions that do not penetrate the muscularis mucosae (Kuiper 1991; Braun et al. 1991; Jensen et al. 1992). Ulcers were classified into 5 types with the following scale: (1) non-perforating ulcers without bleeding, classified into four sub-categories: 1a – small lesions with minimal mucosal defects, 1b – sharply defined ulcer with a punched-out appearance and local mucosal hemorrhage, 1c – ulcer with a superficial coating of detritus (e.g., fibrin or inflammatory products), and 1d – radial wrinkles originating from different directions and converging at a central point or holes through the spiral folds; (2) non-perforating ulcers with arterial bleeding from the left or right gastroepiploic artery; (3) perforated, with acute localized peritonitis; (4) perforated, with diffuse peritonitis; and (5) perforated, with peritonitis and omental bursitis (Braun et al. 1991; Wittek 2022). If the classification of type 1 ulcers to a particular sub-category was not possible, they were classified as ulcer type 1. The ulcer diameter was measured using a ruler. Hyperplasia was defined as the non-neoplastic or neoplastic lesions caused by abnormal growth of tissue from the mucosa (Gelberg 2017). For each abomasum all lesions present were recorded, so one individual could be categorized to more than one group.

### Abomasum parasites

Because of the slaughter procedure, the search and verification of adult forms of parasites were not possible, as all the examined abomasa were already well cleaned. Therefore, the signs of abomasum parasites’ presence in the mucosa as worm nodules and Moroccan leather, were performed by detailed observation of the abomasum. Moroccan leather is a term used to describe the granular appearance of the mucous membrane that can be observed due to mucous cell hyperplasia (Gelberg 2017).

### Statistical analysis

As the variables measured are nominal, the Chi-square test of independence was performed. The statistical analysis was carried out with a significance level established at *P* < 0.05 with the use of the STATISTICA data analysis software system, version 13, produced by TIBCO Software Inc. (2017).

## Results

No pathological findings could be observed in 8 individuals (5.4%). The general results are presented in Tables 2 and 3, and the most important findings are distinguished in the text.

**Table 2:** Lesions found after the inspection of 149 abomasa of Polish fattening bulls in two age groups (bulls less than and more than 2 years old)

	Total (n = 149)		Bulls 1–2-year-old (n = 107)		Bulls older than 2 years old (n = 42)	
Diffuse inflammation						
• Fundus	0	0%	0	0%	0	0%
• Body	95	63.8%	66	61.7%	29	69.0%
• Pylorus	9	6.0%	6	5.6%	3	7.1%
• Whole abomasum	1	0.7%	1	0.9%	0	0%
Erosions						
• Fundus	0	0%	0	0%	0	0%
• Body	80	53.7%	61	57.0%	19	45.2%
• Pylorus	33	22.1%	26	24.3%	7	16.7%
• Whole abomasum	0	0%	0	0%	0	0%
Ulcers						
• 1	10	6.7%	8	7.5%	2	4.8%
1a	21	14.1%	16	15.0%	5	11.9%
1b	37	24.8%	27	25.2%	10	23.8%
1c	1	0.7%	1	0.9%	0	0%
1d	3	2.0%	2	1.9%	1	2.4%
• 2	19	12.8%	11	10.3%	8	19.0%
• 3	0	0%	0	0%	0	0%
• 4	0	0%	0	0%	0	0%
• 5	0	0%	0	0%	0	0%
Ulcers localization						
• Fundus	0	0%	0	0%	0	0%
• Body	29	19.5%	18	16.8%	11	26.2%
• Pylorus	18	12.1%	13	12.1%	5	11.9%
• Body + pylorus	18	12.1%	13	12.1%	5	11.9%
Ulcers scars	17	11.4%	16*	15.0%*	1*	2.4%*
Mucosal adhesions	1	0.7%	1	0.9%	0	0%
Signs of parasites infestation						
• Worm nodules	117	78.5%	87	81.3%	30	71.4%
• Moroccan leather	106	71.1%	79	73.80%	27	64.3%
LDA, RDA	0	0%	0	0%	0	0%
Abomasal volvulus	0	0%	0	0%	0	0%
Abomasal impaction	0	0%	0	0%	0	0%
Neoplasms, hyperplasia	0	0%	0	0%	0	0%
Healthy individuals	8	5.4%	5	4.7%	3	7.1%

*P = < 0.05

LDA: left displacement of the abomasum, RDA: right displacement of the abomasum

In both age groups no signs of inflammation were found in the fundus. The incidence of abomasitis was mostly observed in the body (63.8%), while that of erosions and ulcers mostly seen in the body and pylorus. For bulls < 24 months, only one case of abomasitis was recorded, which was seen on the whole surface of the abomasum. For the occurence of ulceration lesions, type 2 predominated in bulls from the Masovian voivodeship (26.8%), while type 1b was the most prevalent in bulls from Greater Poland (25.5%) (Table 3). The diameter of the lesions varied between 0.2 and 20 cm in older bulls (> 24 months), and between 0.2 and 4 cm in younger bulls (< 24 months). Among the animals with ulcer lesions, 17 (11.4%) had scars after the ulcers healed, and statistically significant differences could be found in their prevalence between the two age groups, with a higher prevalence in younger bulls (*P* = 0.029). Significant differences (*P* < 0.01) were found between the two voivodeships only in terms of the prevalence of type 2 ulcers (26.8% in Masovian, 7.2% in Greater Poland) (Table 3).

**Table 3:** Disorders found after the inspection of 149 abomasa of polish fattening bulls in two voivodeships (Greater Poland and Masovian Voivodeship)

	Total (n = 149)		Greater Poland (n = 109)		Masovian (n = 40)	
Diffuse inflammation						
• Fundus	0	0%	0	0%	0	0%
• Body	95	63.8%	69	63.3%	26	65.0%
• Pylorus	9	6.0%	8	7.3%	1	2.5%
• Whole abomasum	1	0.7%	1	0.9%	0	0%
Erosions						
• Fundus	0	0%	0	0%	0	0%
• Body	80	53.7%	57	52.3%	23	57.5%
• Pylorus	33	22.1%	28	25.7%	5	12.5%
• Whole abomasum	0	0%	0	0%	0	0%
Ulcers						
• 1	10	6.7%	9	8.3%	1	2.5%
1a	21	14.1%	16	14.7%	5	12.5%
1b	37	24.8%	28	25.7%	9	22.5%
1c	1	0.7%	1	0.9%	0	0%
1d	3	2.0%	3	2.8%	0	0%
• 2	19	12.8%	8*	7.4%*	11*	27.5%*
• 3	0	0%	0	0%	0	0%
• 4	0	0%	0	0%	0	0%
• 5	0	0%	0	0%	0	0%
Ulcers localization						
• Fundus	0	0%	0	0%	0	0%
• Body	29	19.5%	20	18.3%	9	22.5%
• Pylorus	18	12.1%	12	11.0%	6	15.0%
• Body + pylorus	18	12.1%	11	10.1%	7	17.5%
Ulcers scars	17	11.4%	17	15.6%	0	0%
Mucosal adhesions	1	0.7%	1	0.9%	0	0%
Sings of parasites infestation						
• Worm nodules	117	78.5%	87	79.8%	30	75.0%
• Moroccan leather	106	71.1%	82	75.20%	24	60.0%
LDA, RDA	0	0%	0	0%	0	0%
Abomasal volvulus	0	0%	0	0%	0	0%
Abomasal impaction	0	0%	0	0%	0	0%
Neoplasms, hyperplasia	0	0%	0	0%	0	0%
Healthy individuals	8	5.4%	4	3.7%	4	10%

*P = < 0.05

LDA: left displacement of the abomasum, RDA: right displacement of the abomasum

## Discussion

Beef breeds in Poland account for 1% of the whole cattle population (Wajda and Burczyk 2013). Due to the trend of breeding for high milk yield and milking traits, milk breed cattle are favoured, and therefore the number of cows that can give birth to mixed-breed calves is limited (Wajda and Burczyk 2013). Therefore, beef production in Poland is currently based largely on dairy breeds, which is why HF bulls constitute the study group in this article.

No pathological position of the abomasum, impaction, volvulus, or hyperplastic lesions were recorded in this study. These observations agree with previous findings, showing that fattening bulls are not a risk group for these disorders (Fubini et al. 2018). However, we cannot fully exclude the possibility that the abomasum returned to its normal position when the animal was slaughtered, chained, and lifted. There are also some rare cases of abomasal impaction in which no dilatation could be observed in *postmortem* examination, and the abomasal content was not fully inspected (Merrit and Boucher 1967). A possible explaination for hyperplasia not being recorded in previous studies could be the young age of the examined animals, and that they were not old enough to show signs of lesion development, or alternatively modern feeding systems did not pose a major threat for gastrointestinal tissue overgrowth.

Most of the examined bulls had inflammatory lesions in the abomasum. The potential consequence of the inflammation process may be the discomfort and pain that can result in decreased feed intake. Moreover, the immune system is activated and requires an increase in glucose intake, which causes increased mobilization of the animal´s energy reserves. The energy demand for an acute case of inflammation is estimated to be > 1 kg of glucose within 720 min (Kvidera et al. 2017). As such, it can be assumed that fattening bulls with these types of lesions do not achieve their maximum body weight, resulting in hidden economic losses. The most common causes of abomasitis and focal inflammation in adult cattle are viral and parasitic diseases (Kuiper 1991). Among the viral factors, bovine viral diarrhea–mucosal disease (BVD-MD), enzootic bovine leukemia (EBL), and bovine malignant catarrhal fever (BMCF) have been the most prevalent reported (Wittek 2022). Since 2017, Poland has been EBL-free, and the cases of BMCF have been sporadic, reported mostly in zoological gardens. On the other hand, a high prevalence of BVD-MD is observed among Polish cattle farms (33.3%). In Greater Poland, the prevalence of BVD virus (BVDV) positive herds is estimated to be 15.3%, while in Masovian Voivodeship the prevalence is even higher, at 39% (Rypuła et al. 2020). Further, cases of more advanced type 2 ulcer lesions were also observed in the Masovian population of cattle, where the virus is more prevalent. This may suggest the involvement of BVDV in the development of abomasum ulceration, but a clear determination of this correlation requires confirmation of the infection by laboratory tests.

While no adult forms of abomasum parasites could be found because of the slaughter procedures, signs of parasites’ presence such as inflammation or worm nodules were observed. These lesions are caused by *Haemonchus, Ostertagia*, and/or *Trichostrongylus* species (Kuiper 1991). This is supported by data from a number of regions in Poland which report invasions of the abomasum caused by *Ostertagia*, and *Trichostrongyloides* (Tomczuk et al. 2018). In this study, 71.1% of the animals had lesions described as Moroccan leather associated with *Ostertagia* invasion (Gelberg 2017), and even more individuals had worm nodules, which shows that endoparasites can be a marginalized problem. These high infestation rates may be due to farmers not providing deworming prophylaxes to their herds, or that the drugs currently available may no longer be effective. In Poland, the most common means of treatment is injection of the antiparasitic drug ivermectin, due to its low cost and high efficiency against ecto- and endoparasites (Mickiewicz et al. 2021). However, in a number of countries, including Germany, Belgium and Sweden, complete resistance of *Ostertagia ostertagii* to ivermectin has been reported on some farms (Demeler et al. 2009). Similarly, resistance to benzimidazoles, another popular anthelmintic drug used to treat parasitic worms in Poland, has recently been reported in the United Kingdom (Bartley et al. 2021; Mickiewicz et al. 2021). This suggests that even if the correct prophylaxe protocols are followed, the drugs may no longer be effective, and that the increasing reports of drug resistance from other European countries may also be indicative of resistance occurring in Poland. Currently, data on parasite resistance in Poland only comes from Polish goats’ herds, where wide spread resistance by *H. contortus* and *Trichostrongylus* spp. to macrolytic lactones and benzimidazoles has been reported (Mickiewicz et al. 2021). Current treatments rely on the antihelminthic drug levamisole, which has been found to be the most effective drug (Mickiewicz et al. 2021).

Ulcers of the abomasum in bulls are of multifactorial origin (Fubini et al. 2018). They may appear occasionally after transport, long-term non-steroid drugs/glucocorticoids application, or painful incidents/surgeries. Up to 33% of yearling feedlot cattle may be affected by grain overdose, at all stages of fattening (Jensen et al. 1992). For macroscopical evaluation of ulcers the adopted scale is widely used, and its acceptable reliability is proven (Van Driessche et al. 2023). In Austria, only type 1 ulcers were found in the abomasum of 65.9% of the examined bulls, mainly in the pylorus (63.3%) (Hund et al. 2016). In Danish dairy cows (84% of HF breed), subtype 1c constituted the highest prevalence (34%), and one case of type 3 was reported (Munch et al. 2019). In the present study, type 1 constituted the largest percentage in Greater Poland, while in Masovian more advanced lesions of type 2 ulcers were dominant. These lesions were probably associated only with a small degree of intraluminal hemorrhage with no clinical signs observed (Kuiper 1991). A more chronic course could be suspected in the case of animals from Greater Poland as wounds after healing and one case of mucous membrane adhesions could be observed. One of the most probable reasons for the development of ulcers could be the feeding system, which is focused on increasing the maximum growth rate in the last month before slaughter. Most fattening bulls in Poland are kept in closed barns, in a semi-intensive system (Nogalski and Wroński 2011). The use of total-mixed ration (TMR) is popular in these feeding systems, although the inclusion of poor-quality silage could be a causal reason for the development of ulcers (Wójcik 2011).

## Conclusions

It can be assumed that the fattening bulls’ management system in Poland favours the formation of low-grade abomasum ulcers and erosions. Even though animals are reared in conditions conducive to good growth, many of them develop subclinical inflammation of the abomasum, which results in a decrease in production and economic losses, as well as being a welfare issue. Further research should be performed to determine the specific factors that cause abomasal lesions, including feeding and housing systems.

### Electronic supplementary material

Below is the link to the electronic supplementary material.

Supplementary Material 1

## Data Availability

No datasets were generated or analysed during the current study.
