# Supplementary material for: Abomasal lesions found in postmortem examination of fattening Holstein-Friesian bulls from central Poland
Source: Vet Res Commun. 2024 Mar 28;48(3):1963–9. doi: 10.1007/s11259-024-10366-4 (PMC11147930; doi:10.1007/s11259-024-10366-4)
Supplement: Supplementary file 1 — Supplementary Material 1 [file 11259_2024_10366_MOESM1_ESM.docx]

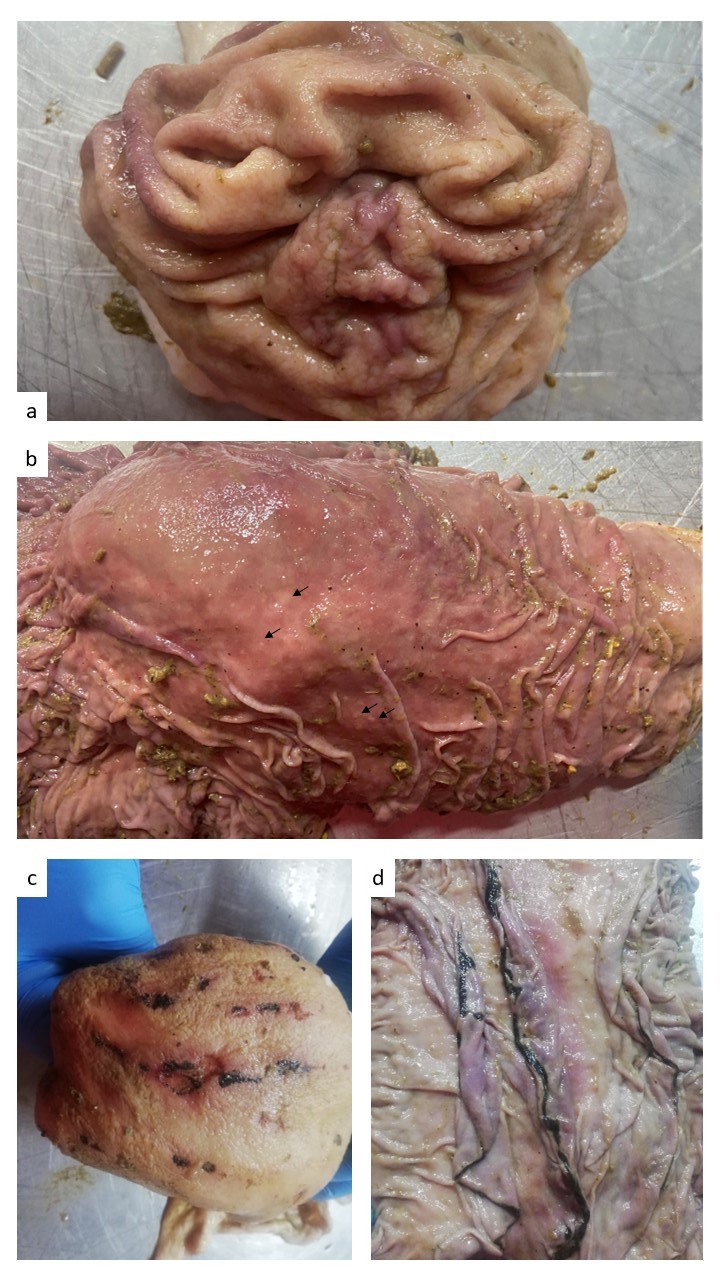


Figure 1 – Examples of the lesions found during *postmortem* inspection of 149 examined Holstein-Friesian bulls: a – Moroccan leather, b – worm nodules (some of them marked with arrows) with inflammation of the abomasa wall, c – ulcer type 2 with inflammation lesions, d – 20 cm ulcer type 1b
